# Transient anterior subcapsular vacuolar lens opacities after Tanito microhook trabeculotomy: report of six cases

**DOI:** 10.1186/s12886-024-03498-3

**Published:** 2024-05-29

**Authors:** Hiroshi Shimizu, Masaki Tanito

**Affiliations:** 1 Meizankai Shimizu eye clinic, Matsue, Japan; 2 Department of Ophthalmology, Faculty of Medicine, Shimane University, Izumo, Japan

**Keywords:** Tanito Microhook trabeculotomy (TMH), Anterior subcapsular cataract (ASC), Minimally invasive glaucoma surgery (MIGS), Surgical complication, Transient cataract

## Abstract

**Objective:**

To present six cases exhibiting transient anterior subcapsular vacuolar lens opacities following early postoperative Tanito microhook trabeculotomy (TMH) performed by the same surgeon.

**Methods:**

Six patients who underwent lens-sparing TMH at Meizankai Shimizu Eye Clinic from November 2021 to May 2023, and developed anterior subcapsular vacuolar lens opacities postoperatively were reviewed. Detailed records of surgeries, follow-up findings were collected and reported.

**Results:**

In all six cases, anterior vacuolar subcapsular lens opacities were observed on the day after surgery, gradually decreasing without affecting visual acuity or contrast sensitivity. In all cases, without any specific interventions, the opacities disappeared by 21 months postoperatively.

**Conclusion:**

Anterior subcapsular cataracts, characterized by a vacuolar appearance and transient existence, should be recognized as an early complication of ab interno glaucoma surgery, possibly linked to use of distributed ophthalmic viscosurgical devices and excessive anterior chamber irrigation leading to traumatic cataracts on the lens surface.

Tanito microhook trabeculotomy (TMH), introduced in 2016 as a minimally invasive glaucoma surgery (MIGS) for managing glaucoma [1], has gained wide adoption since its inception. Reported postoperative complications of TMH encompass anterior chamber hemorrhage [2, 3], ciliary body detachment [4–7], anterior iris adhesion [8], and corneal hypermetropia [9]. While cataracts have been reported as a long-term complication in cases preserving the lens [3], early postoperative cataracts have not been documented. This report describes characteristic early postoperative vacuolar cataracts under the anterior capsule of the lens in six consecutive cases of TMH performed by the same surgeon.

## Methods

This case series encompasses six consecutive cases of transient anterior subcapsular vacuolar lens opacities post TMH performed by the same surgeon (H.S.) at Meizankai Shimizu Eye Clinic between November 2021 and May 2023. A meticulous review of all surgical records was conducted, encompassing comprehensive report of clinical observations and outcomes.

The same surgical technique was performed in all cases. Preoperatively, pilocarpine hydrochloride drops (Sanpilo 2%, Santen Pharmaceutical, Osaka, Japan) were administered for anterior pupillary constriction. The ocular surface was prepped using povidone-iodine (Isodine solution 10%, Mundipharma K.K., Tokyo, Japan), followed by application of disposable sterilized surgical eye drapes. Conjunctival tissues were cleansed with iodine polyvinyl alcohol (PA-Iodo Ophthalmic and Eye Washing Solution, Nitten Pharmaceutical Co. Ltd., Nagoya, Japan), while sub-Tenon injection of lidocaine (Xylocaine 4%, Sandoz Pharma K.K., Tokyo, Japan) was employed for topical anesthesia induction; no intracameral anesthesia was administered. Subsequent to the creation of side ports at 2 and 10 o'clock positions, a viscoelastic material comprising purified sodium hyaluronate and chondroitin sulfate sodium (Viscoat, Alcon, Japan) was introduced into the anterior chamber. Ab interno trabeculotomy was executed employing microhooks (Inami & Co., Ltd., Tokyo, Japan) for incising Schlemm's canal and trabecular meshwork over a 3 clock-hour span. Post-trabeculotomy, the viscoelastic material was evacuated via bilateral side ports utilizing the Constellation (Alcon, Japan) along with 23G bimanual handpieces (Sterimedix Ltd., Redditch, UK), accompanied by balanced salt solution (BSS Plus, Alcon, Japan) as the irrigating fluid. Irrigation pressure was maintained at 80 cmH_2_O, with a maximum aspiration pressure of 400 mmHg and a maximum aspiration flow rate of 30 cc/min to prevent anterior chamber collapse. Closure of side ports was achieved through corneal stromal hydration. To minimize the bleeding from the trabeculotomy cite, the surgery was completed with increased IOP. At the end of surgery, subconjunctival injection of 2 mg betamethasone sodium phosphate (Rinderon, Shionogi Pharma Co. Ltd., Osaka, Japan) was performed, followed by application of 0.3% ofloxacin ointment (Tarivid, Santen Pharmaceutical). Subsequently, topical applications of 1.5% levofloxacin (Cravit, Santen Pharmaceutical) and 0.1% betamethasone (Rinderon, Shionogi Pharma Co. Ltd., Osaka, Japan) were administered three times daily for three weeks.

## Results

Table 1 summarize the details of the cases.

**Table 1:** Summary of cases

**Case No.**	**Age**	**Sex**	**Diagnosis**	**Preoparative** **Refraction (Diopter)**	**Surgical Time (minutes)**	**Maximal intraoperative mydriasis** **(mm)**	**Subcapsular opacity** **At POD1**	**Subcapsular opacity** **At final visit**	**Preoperative BCVA (decimal)**	**BCVA at POD1 (decimal)**	**BCVA at final visit (decimal)**	**Follow-up period after operation (months)**
1	59	M	POAG	-6.00	11	7	+++	+	1.0	0.9	1.0	9
2	47	F	POAG	-8.75	12	6	++	+	1.0	0.8	1.0	12
3	48	F	POAG	-1.75	14	4	++	+	1.0	1.0	1.0	21
4	68	M	POAG	-0.50	14	3	+	-	1.0	1.0	1.0	10
5	59	F	POAG	-0.75	12	5	++	-	1.0	1.0	1.0	18
6	59	F	POAG	-7.0	8	5	++	+	1.0	0.9	1.0	15

*M* Male, *F* Female, *POAG* Primary open angle glaucoma, *BCVA* Best-corrected visual acuity

**Fig. 1:**
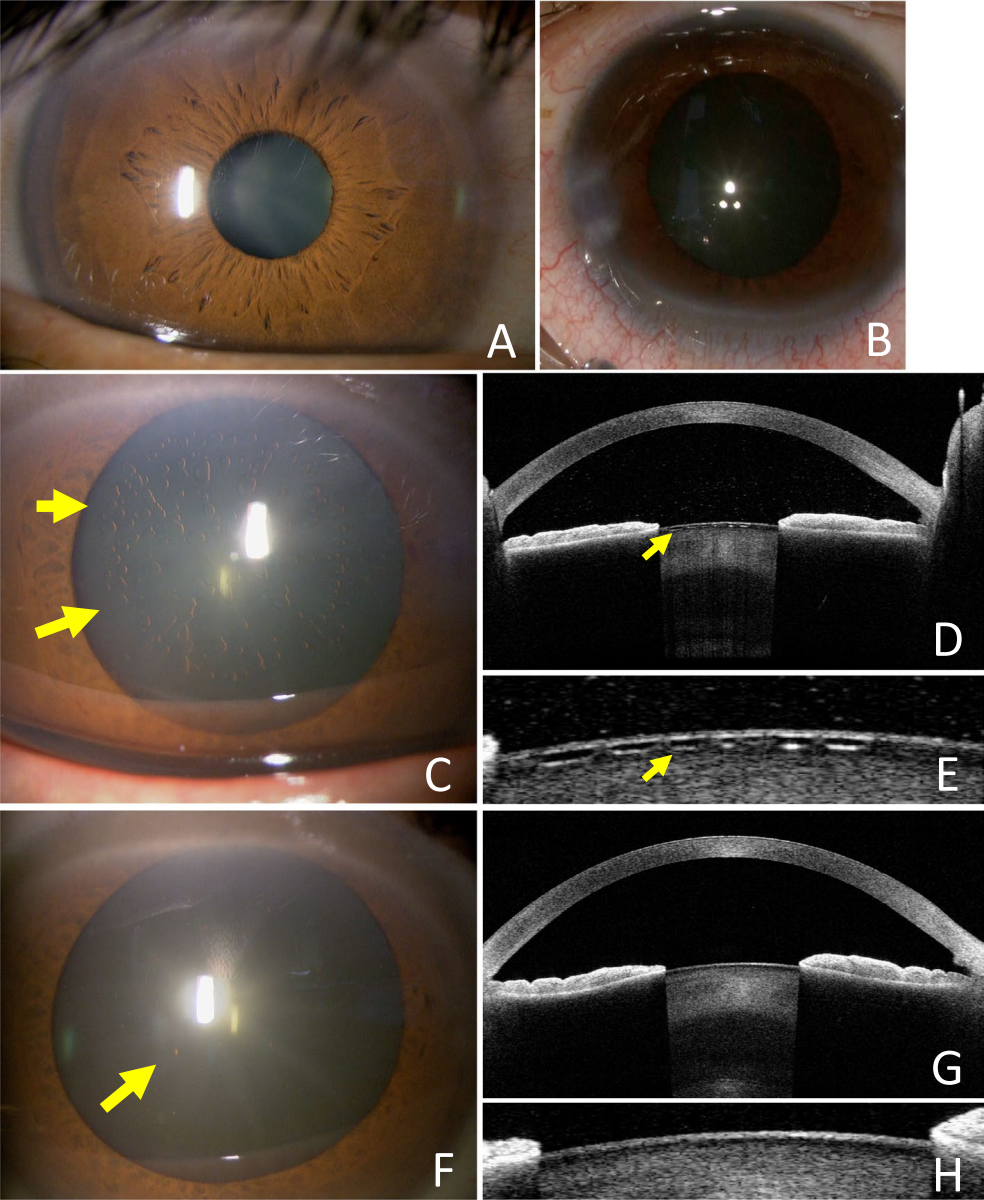
Case 1. A Slit-lamp photographs and AS-OCT images. No preoperative anterior subcapsular cataract (**A**). Mydriasis increases to a maximum of about 7 mm as intraoperative IOP increased (**B**). POD1 showed vacuolar anterior subcapsular opacities of the lens (arrows) within 6 mm of the pupil center (**C**). Opacities are observed beneath the anterior lens capsule (arrow) on AS-OCT (**D**), and observed as small cysts with inner hypointense and outer hyperintense signals (arrow) in the magnified image (**E**). Nine months after surgery, the opacity had almost disappeared, but some remained (arrow) (**F**). cysts on AS-OCT had disappeared (**G**, **H**)

Case 1 was a 59-year-old man who underwent TMH for primary open-angle glaucoma (POAG) in the right eye, which did not have an anterior subcapsular cataract preoperatively (Fig. 1A). At the beginning of the surgery, pupil diameter was about 3 mm. When Viscoat was replaced in the anterior chamber before trabeculotomy, pupil was dilated to about 5 mm because of the elevated IOP. After trabeculotomy, irrigation and aspiration were performed through the side port bimanually using a Constellation. When the irrigation handpieces were removed, reflux hemorrhage occurred with anterior chamber collapse, so the wound was hydrated, and irrigation aspiration was performed again; pupil was dilated up to about 7 mm with the increase of IOP (Fig. 1B). The operative time was 11 minutes. On postoperative day (POD) 1, there was mild anterior chamber hemorrhage, and best-corrected visual acuity (BCVA) was 0.9 in decimal. Subcapsular vacuoles lens opacity was distributed over a 6 mm area at the center of the pupil (Fig. 1C). Anterior segment optical coherence tomography (AS-OCT, Casia 2, Tomey Corporation, Nagoya, Japan) showed cystic structures with inner hypo-reflective and outer hyper-reflective signals just beneath the anterior lens capsule, corresponding to the vacuolar lens opacities (Fig. 1D, E). The patient had no subjective symptoms, and his BCVA recovered 1.0 as the anterior chamber hemorrhage resolved. Subcapsular vacuoles decreased each passing month, and almost disappeared after nine months (Fig. 1F). Cysts had disappeared in AS-OCT (Fig. 1G, H).

**Fig. 2:**
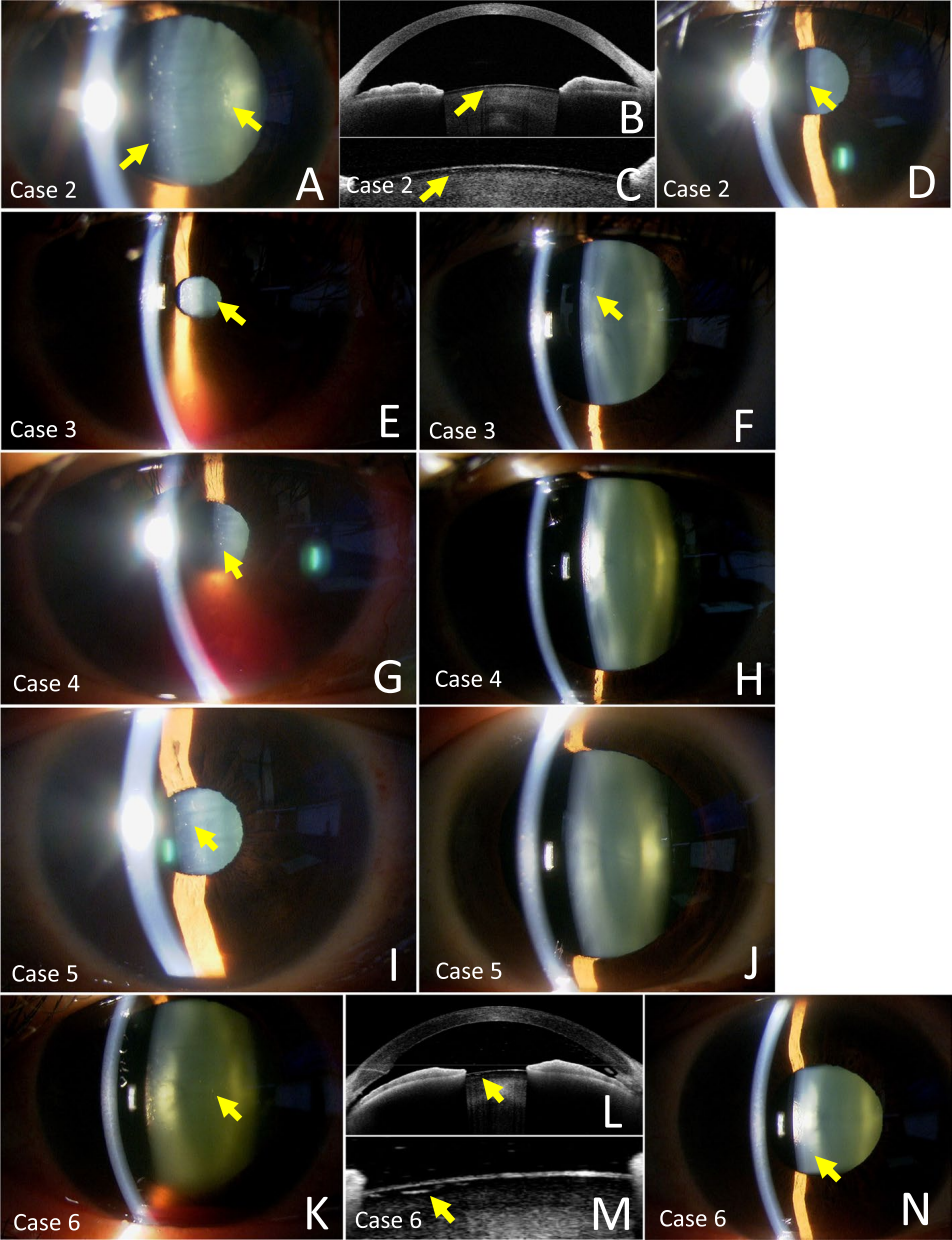
Slit-lamp photographs of POD1 (**A**, **E**, **G**, **I**, **K**), early postoperative AS-OCT images (**B**, **C**, **L**, **M**) and slit-lamp photographs during later follow-up periods (**D**, **F**, **H**, **J**, **N**) of Cases 2-6,. All cases showed subcapsular vacuolar opacities under the anterior lens (arrows) as in Case 1 (**A**, **E**, **G**, **I**, **K**). Cases 2 and 6 showed cysts under the anterior capsule (arrows) on AS-OCT similar to Case 1 (**B**, **C**, **L**, **M**). After the midterm postoperative periods, Cases 4 and 5 show almost complete disappearance of opacity (**H**, **J**), while Cases 2, 3, and 6 show a decrease of opacities (arrows) (**D**, **F**, **N**)

Case 2 was a 47-year-old woman who underwent TMH for POAG of the right eye. The surgery was performed in the same manner as Case 1, a maximum intraoperative mydriasis of 6 mm, and an operative time of 11 minutes. On POD1, a characteristic anterior subcapsular cataract a were observed (Fig. 2A). AS-OCT also showed an internal hypointense void (Fig. 2B, C). There were no subjective symptoms, and the BCVA was 1.0 preoperatively and 1.0 postoperatively. Twelve months after surgery, the opacity had decreased (Fig. 2D).

Case 3 was a 48-year-old male who underwent TMH for POAG of the right eye. The maximum intraoperative mydriasis was 4 mm, and the operative time was 14 minutes. On the POD1, a slight but characteristic anterior subcapsular cataract cataract was observed, with a mild anterior chamber hemorrhage (Fig. 2E), with no loss of vision, no subjective symptoms, and a BCVA of 1.0 preoperatively and 1.0 postoperatively. Twenty-one months after surgery, the opacity had decreased. (Fig. 2F).

Case 4 is a 68-year-old woman who underwent TMH for POAG of the right eye. The maximum intraoperative mydriasis was 3 mm, and the operative time was 14 minutes. On POD1, a similar cataract was observed the day after surgery with moderate anterior chamber hemorrhage (Fig. 2G), with no loss of vision, no subjective symptoms, and a BCVA of 1.0 preoperatively and 1.0 postoperatively. Ten months after surgery, the anterior subcapsular cataract had almost disappeared (Fig. 2H).

Case 5 was a 59-year-old woman who underwent microhook trabeculotomy for POAG of the left eye. The maximum intraoperative mydriasis was 5 mm, and the operative time was 12 minutes. On the POD1, a very small amount of characteristic anterior subcapsular cataract was observed (Fig. 2I), and the BCVA was 1.0 preoperatively and 1.0 postoperatively. Eighteen months after surgery, the opacity had almost disappeared (Fig. 2J).

Case 6 was a 59-year-old woman who underwent TMH for POAG in the right eye. The maximum intraoperative mydriasis was 5 mm, and the operative time was 8 minutes. The day after surgery, there was a hyphema and a characteristic anterior subcapsular cataract (Fig. 2K), AS-OCT also showed an internal hypointense void (Fig. 2L, M). There were no subjective symptoms, and the BCVA of 1.0 preoperatively and 1.0 postoperatively. Three months after surgery, the opacity had decreased (Fig. 2N).

## Discussion

All six consecutive cases of lens-sparing TMH performed by H.S. resulted in distinct cataracts observed on POD1. Notably, H.S. possesses extensive experience in ophthalmic surgery, particularly in MIGS, and during these procedures, there was no direct contact between surgical instruments and the lens surface, as evidenced in the video documentation. Additionally, the circular and vacuolar nature of the observed opacities likely did not indicate a traumatic cataract resulting from direct instrument contact. These circular vacuoles appeared within the range of intraoperative mydriasis, spanning from 3-7 mm (refer to Table 1), notably peaking during irrigation-aspiration maneuvers across all cases.

This suggests considerable elevation of IOP, possibly leading to substantial water flow impacting the lens surface. A review of 560 TMH cases, including 80 lens-sparing procedures, conducted by the procedure's developer, reported six postoperative cataracts over an average follow-up of 405 days [3]. However, the characteristic cataracts observed in the very early postoperative period have not been reported in TMH procedures to date.

Disparities in technique between TMH described in previous report [3] and TMH performed by H.S. in the current report are detailed in Table 2. For instance, while in previously reported TMH employed cohesive OVD (Opegan High, Provisc), H.S. opts for dispersive OVD (Viscoat) requiring more extended removal time. Additionally, TMH concluded with a moderate IOP, whereas H.S. maintained a slightly elevated IOP post-surgery to prevent reflux hemorrhage. This difference in surgical approach suggests three possible mechanisms for the development of the anterior subcapsular cataract in these cases.

**Table 2:** Comparison of drugs, surgical equipment, and techniques used in previously reported TMH and TMH performed by H.S

	**Previously reported TMH**	**H.S.’s TMH**
Preoperative medications	Sanpilo 2% Levofloxacin 1.5%	Sanpilo 2% Cravit 1.5%
Anesthetics	Xylocaine 1%(intracameral) Benoxil Ophthalmic Solution 0.4%	Xylocaine 4%(drop)
Surgical instrument	Centurion	Constellation
Infusion fluid	BSS Plus	BSS Plus
Viscoelastic material	Opegan Hi Provisc	Viscoat
Irrigation and aspiration handpiece	Centurion Irrigation and Aspiration handpiece (Alcon Japan, Tokyo, Japan)	23G bimanual handpiece (Sterimedix Ltd., Redditch, UK)
Range of trabeculotomy	200	200
IOP at the end of surgery	Moderate	High
Subconjuctival injection	Decadron	Rinderon
Ointment	Tarivid	Tarivid

Sanpilo 2%(Santen Pharmaceutical , Osaka, Japan), pilocarpine hydrochloride

Cravit(Santen Pharmaceutical), 1.5% levofloxacin

Levofloxacin (Pfizer Japan Inc., Tokyo, Japan), 1.5% levofloxacin

Xylocaine1% (Aspen Japan, Tokyo, Japan), lidocaine

Benoxil ophthalmic solution 0.4% (Santen Pharmaceutical), oxybuprocaine hydrochloride

Xylocaine 4% (Aspen Japan, Tokyo, Japan), lidocaine

BSS Plus (Alcon Japan, Tokyo, Japan), balanced salt solution

Opegan Hi (Santen Pharmaceutical), Provisc (Alcon Japan), purified sodium hyaluronate

Viscoat (Alcon Japan), purified sodium hyaluronate and chondroitin sulfate sodium

Decadron (Aspen Japan), dexamethasone sodium phosphate

Rinderon (Shionogi Pharma Co. Ltd., Osaka, Japan), betamethasone sodium phosphate

Tarivid (Santen Pharmaceutical), 0.3% ofloxacin ointment

The first hypothesis is due to differences in the nature of the selected OVDs. The dispersed OVD used in these cases has stronger tissue adhesion and hence higher intraocular retention than the cohesive OVD used in previous reports. [10–12]. In modern cataract surgery, the lens capsule and lens cortex can be readily separated during capsulorrhexis and hydrodissection due to the fact that the capsule originates from the basement membrane of the epithelial cells of the embryonic lens vesicle. We hypothesize that vacuoles are formed when a strongly adhered dispersed OVD on the anterior capsule is forcibly detached in the anterior direction during IA manipulation, resulting in the separation of the anterior chamber and the crystalline lens.

The second possible mechanism is forceful irrigation in anterior chamber. Another procedure involving lens-sparing manipulation in anterior chamber, implantable collamer lens (ICL) insertion surgery, is recognized for its association with cataract development, particularly in older and more myopic patients. Reports indicate that forceful irrigation post ICL insertion induced cataracts [13–19], showcasing similarities with our observations in TMH. The mechanism appears akin to anterior subcapsular opacity formation due to water flow impacting the anterior lens surface during OVD removal.

The third hypothesis is due to the I/A procedure during the surgery, the elevated IOP post-surgery might also contribute to the occurrence of the anterior subcapsular lens opacities. The Glaukomflecken sign is known as a classic yet uncommon indicator following an acute angle-closure attack. It manifests as subcapsular fleck-like opacity resulting from necrosis of the lens epithelium due to elevated intraocular pressure. Opacities observed in this case, being vacuolar rather than fleck-like, and diminishing over time, renders this hypothesis less plausible.

Plausible alternative diagnoses such as direct instrument contact, drug toxicity, or perioperative factors were considered but dismissed. This report represents the first documentation of early postoperative cataracts following TMH. While vision remained unaffected in our case series, severe opacities could potentially impact vision.

To avoid such complications, we recommend using cohesive rather than dispersive ophthalmic viscosurgical devices (OVDs), ensuring that the irrigation flow during IA procedure does not directly contact the anterior capsule, and avoiding excessive elevation of intraocular pressure. In cases where these two measures were implemented, subsequent occurrences of similar subcapsular opacity have not been observed. As such, meticulous care during irrigation techniques is recommended in lens-sparing surgeries within the realm of ab interno MIGS, not limited to TMH.

## Data Availability

No datasets were generated or analysed during the current study.

## Abbreviations

TMH: Tanito microhook trabeculotomy
POD: Postoperative day
IOP: Intraocular pressure
AS-OCT: Anterior segment optical coherence tomography
BCVA: Best-corrected visual acuity
MIGS: Minimally invasive glaucoma surgery
OVD: Ophthalmic viscosurgical devices
ICL: Implantable Collamer Lens
